# Diagnostic performance of contrast-enhanced voiding ultrasonography and direct radionuclide cystography with physiologic bladder filling volumes in pediatric vesicoureteral reflux—a prospective study

**DOI:** 10.1007/s00431-026-06814-9

**Published:** 2026-03-15

**Authors:** Hanna-Reeta Viljamaa, Tiina Laurikainen, Niklas A. Pakkasjärvi, Marko Seppänen, Päivi T. K. Rautava, Liisi L. M. Ripatti

**Affiliations:** 1 Department of Pediatric Surgery, Turku University Hospital and The Wellbeing Services County of Southwest Finland, Turku, Finland; 2 University of Turku Finland, Turku, Finland; 3 Department of Diagnostic Radiology, Turku University Hospital and The Wellbeing Services County of Southwest Finland, Turku, Finland; 4 Department of Pediatric Surgery, New Children’s Hospital, Helsinki University Hospital and University of Helsinki, Stenbäckinkatu 9, PL 347, 00029 Helsinki, Finland; 5 Faculty of Medicine, Vaasa Central Hospital and University of Turku, Turku, Finland; 6 Department of Clinical Physiology, Nuclear Medicine and Turku PET Centre and Department of Medical Physics, Turku University Hospital and The Wellbeing Services County of Southwest Finland, Turku, Finland; 7 Department of Public Health, University of Turku and Turku Clinical Research Centre, Turku University Hospital and The Wellbeing Services County of Southwest Finland, Turku, Finland

**Keywords:** VUR, DRC, CEVUS

## Abstract

To compare the diagnostic performance of contrast-enhanced voiding ultrasonography (CEVUS) and direct radionuclide cystography (DRC) at physiological bladder filling volumes in pediatric patients with suspected vesicoureteral reflux (VUR). This prospective, comparative study included 22 children with 44 renal units referred for evaluation due to recurrent urinary tract infections (UTIs). Bladder function was assessed as normal through a four-hour voiding observation or uroflowmetry. All patients underwent both DRC and CEVUS on the same day, using a single catheterization. The investigations were performed by blinded radiologists. The primary outcome was VUR detection in relation to bladder filling volumes. Secondary outcomes included adverse effects and clinical outcomes in relation to radiological findings. DRC detected VUR in 72.7% of patients and 50.0% of renal units, while CEVUS detected VUR in 77.3% and 52.3% respectively. Among patients with paired bladder-filling data at first VUR detection, median bladder filling volume was 37.3% (IQR 26.7–82.4) of expected bladder capacity for DRC and 66.7% (IQR 52.9–87.3) for CEVUS (Wilcoxon signed-rank *p* = 0.158). At patient level, paired detection rates were comparable between modalities (exact McNemar *p* = 1.00). At the renal unit level, intermodality agreement was moderate (Cohen’s *κ* = 0.59; 95% CI 0.35–0.83). DRC detected VUR in 4 renal units negative on CEVUS, whereas CEVUS detected VUR in 5 renal units negative on DRC.

*Conclusion*: CEVUS was comparable to DRC in VUR detection with physiological bladder filling volumes. As a radiation-free method providing anatomical detail while minimizing bladder overfilling, CEVUS offers a valuable alternative to VUR imaging.

**Table:** 

**What is Known:** • *Voiding cystourethrography and direct radionuclide cystography are established imaging modalities for diagnosing vesicoureteral reflux, but both involve ionizing radiation.*
**What is New:** • *Radiation-free contrast-enhanced voiding ultrasonography detects vesicoureteral reflux using physiologic bladder filling volumes with detection rates comparable to direct radionuclide cystography.*

**What is Known:**

• *Voiding cystourethrography and direct radionuclide cystography are established imaging modalities for diagnosing vesicoureteral reflux, but both involve ionizing radiation.*

**What is New:**

• *Radiation-free contrast-enhanced voiding ultrasonography detects vesicoureteral reflux using physiologic bladder filling volumes with detection rates comparable to direct radionuclide cystography.*

## Introduction

Vesicoureteral reflux (VUR) is the retrograde flow of urine from the bladder into the ureter(s) due to inefficient ureteral action and a deficient closure mechanism at the ureterovesical junction, often compounded by dysfunctional bladder dynamics [1]. In children with febrile urinary tract infections (UTI), the prevalence of VUR is 35%, but only 0.4 to 1.8% in those with no history of UTIs [2–4]. Most guidelines recommend evaluating for VUR following recurrent or atypical UTIs [5–7]

Voiding cystourethrography (VCUG) is the standard diagnostic tool for VUR but involves ionizing radiation [5]. Effective radiation exposure from VCUG ranges 0.03–0.3 mSv, depending on the equipment used [8]. Direct radionuclide cystography (DRC) was introduced to reduce radiation exposure, with sensitivity ranging from 71 to 100% and specificity from 67 to 100%, similar to VCUG [9]. Additionally, DRC offers the advantage of reduced bladder filling volumes but lacks the anatomical detail that VCUG provides. Optimization protocols for VCUG have subsequently lowered its radiation doses below those of DRC [10].

Recent developments in ultrasound contrast agents (UCAs) have enabled the detection of VUR without ionizing radiation [11, 12]. Second-generation UCAs like SonoVue® allow contrast-enhanced voiding ultrasonography (CEVUS) to achieve high sensitivity (97%) and specificity (93%) for VUR detection [13]. CEVUS demonstrates excellent agreement with VCUG, particularly for detecting grade 2 VUR and above [14–19] Additionally, intrarenal reflux (IRR) is reportedly more frequently detected with CEVUS than with VCUG [20, 21] However, the clinical significance and optimal assessment of IRR remain incompletely defined. CEVUS also provides anatomical detailing, enabling the identification of proximal tract dilatation in contrast to DRC.

VUR may occur intermittently, necessitating at least two voiding cycles during VCUG [22]. However, bladders in children undergoing VCUG are often overfilled, with volumes exceeding age-adjusted bladder capacity by an average of 31% [23]. This overfilling may lead to overdiagnosis and subsequent unnecessary interventions due to iatrogenic reflux.

In this context, we investigated whether DRC and CEVUS share comparable sensitivity for detecting VUR at physiologic bladder filling volumes. Specifically, we explored whether CEVUS provides sufficient sensitivity for accurate VUR diagnosis, potentially offering an optimized diagnostic pathway compared to DRC. The primary outcome was identification of VUR. Secondary outcomes included any adverse events with the imaging modalities, and short-term clinical outcomes. This prospective study involved blinded investigators for DRC and CEVUS with a single catheterization to ensure objective and independent evaluations.

## Materials and methods

We enrolled patients referred to pediatric urology at a tertiary university hospital for evaluation due to recurring urinary tract infections between May 2022 and May 2025. Recurrent UTI is defined as at least three episodes of UTIs per year or two episodes of UTIs in the past 6 months [5]. Oral and written information was provided and written informed parental consent was obtained.

Inclusion criteria were age under 6 years, at least three episodes of UTIs per year or two episodes of UTIs in the past 6 months and normal voiding and bladder function. Exclusion criteria included abnormal bladder function or voiding, clinical suspicion or previous history of posterior urethral valve, parental refusal, and hypersensitivity to the active substances or excipients of the investigational products. Bladder function was assessed in infants by 4-h voiding observation and in older children via uroflowmetry, adhering to International Children’s Continence Society principles for the evaluation of normal voiding function. Residual urine volume was determined by ultrasound immediately after urination by a urotherapist. Normal expected age-standardized bladder capacity (EBC) was calculated using the Koff formula: bladder capacity (ml) = (age in years + 2) × 30 ml [24]. Physiological bladder filling was defined as filling not exceeding the EBC. Bladder emptying was considered normal when residual urine volume was less than 20% of EBC.

All patients underwent both CEVUS and DRC on the same day and all underwent CEVUS first. A 6 Fr urinary catheter without inflatable balloon was placed in the bladder by a urotherapist or pediatric radiologist. The same catheter was used for both CEVUS and DRC. The bladder was filled to EBC and the filling rate was standardized using a drop counter, allowing slow filling. During filling, patients were continuously observed for leakage, discomfort, or urge to void, and filling was interrupted if clinically indicated. In both CEVUS and DRC, patients underwent two separate filling–voiding cycles, and the bladder was emptied via catheter in between. CEVUS investigations were performed by a pediatric radiologists and DRC by a nuclear medicine physician. Independent CEVUS and DRC investigators were blinded to other imaging modality data. Diagnostic accuracy metrics are reported relative to DRC as the comparator and should be interpreted as measures of agreement rather than absolute accuracy versus an independent gold standard.

Siemens Acuson Sequoia ultrasound equipment with C5-1 convex array transducer was used for all examinations. Before catheterization a conventional urinary tract ultrasound evaluation of the bladder, ureters and kidneys was performed. After catheterization, the bladder was emptied. UCA (SonoVue® [Bracco, Milan, Italy]) was initially administered into a plastic bag of 0.9% normal saline. A 0.2% contrast/saline solution was mixed. The solution was administered as a 5-min infusion. Homogenous contrast material distribution in the bladder was evaluated with repeated ultrasound evaluations using dedicated software and low mechanical index. Additional 2% SonoVue boluses were administered, if needed, to maintain appropriate contrast in the bladder. The ureters and kidneys were evaluated during and after the filling and voiding phase. Reflux was graded 1–5 according to guidelines [25].

During DRC, the child was supine on the gamma camera (Discovery NM/CT 870 DR, GE Healthcare). The bladder was first emptied through the same catheter inserted for CEVUS. A radiotracer (99mTc-pertechnetate, 20 MBq) was then introduced into the bladder, followed by a 5-min infusion of Sodium chloride 9 mg/ml warmed to body temperature. Sequential images were acquired during both bladder filling and voiding phases. Any observed radiotracer activity in the pelvis or ureteral regions was considered positive for VUR.

Parents were instructed to offer their children habitual food and drink before the examinations. Afterwards, we recommended that the child drink ample fluids and urinate more frequently to flush the bladder of the radionuclide used in the DRC. Each study participant was given a prophylactic antibiotic (trimetoprine 2–3 mg/kg or nitrofurantoine 1–2 mg/kg) for at least three days after examinations. If prophylactic antibiotics had been used before the examinations, the dose was doubled for 3 days. Thereafter, the previous dose was continued.

From 2023, the respective costs for DRC in our hospital were 679 euros and CEVUS 213 euros. Since cost data are institution specific, these costs are indicative.

### Statistical analyses

Data were summarized using descriptive statistics and presented as medians with interquartile ranges (IQRs) given the limited sample size. Normality was assessed visually and by the Shapiro–Wilk test. Bladder filling volumes at the time of VUR detection were compared between modalities within patients using the Wilcoxon signed-rank test based on the patient-level first-detection bladder filling volume (%EBC) for each modality. Patient with missing bladder-filling volume at VUR detection were excluded from the bladder-filling comparison but retained for categorical detection analyses.

Given the paired, agreement-based study design, CEVUS was compared with DRC as the clinical comparator; VCUG, although the standard reference test in clinical practice, was not added solely for research purposes to avoid additional ionizing radiation exposure. Paired differences in patient level VUR detection between modalities were assessed using McNemar’s test. Inter-modality agreement was evaluated using Cohen’s kappa at the renal unit level. For comparative purposes, diagnostic accuracy measures (sensitivity, specificity, positive and negative predictive value, and likelihood ratios) were calculated for CEVUS using DRC as the reference comparator and should be interpreted as measures of agreement rather than absolute accuracy versus an independent gold standard.

#### Unit of analysis and within-patient correlation

Primary inferential analyses were performed at patient level to avoid within-patient correlation between renal units. Patient-level VUR status was defined as presence of VUR in either renal unit on a given modality. Renal unit-level results are presented as supportive data to describe laterality, unit-level concordance, and discrepant findings.

All tests were two-sided. *p*-values < 0.05 were considered statistically significant. Analyses were performed using Stata/BE (StataCorp LLC, College Station, TX, USA).

### Ethics

This study was approved by the Hospital District of Southwest Finland Ethics Committee (ETMK121/2121) and registered at Clinical Trials (EudraCT 2021–006371-42). It was also approved by the Finnish Medicines Agency (KLnro 148/2021) and registered in the Clinical Trial Information System (CTIS). The study was conducted in accordance with the Declaration of Helsinki.

## Results

We screened 22 patients under 6 years of age with at least two culture-positive UTIs. All 22 patients, 15 girls (68.2%), and seven boys (31.8%), corresponding to 44 renal units, met the inclusion criteria. Median age at enrolment was 0.9 years (IQR 0.6–3.1 years). Seven patients (33%) had urinary tract anomalies (six with unilateral and one with bilateral duplex systems) and one patient had Turner’s syndrome. The median number of UTIs before participation was three (range 2–5). In 20 (90.9%) patients, UTIs were febrile. *E. coli* was isolated in 20 (90.9%) patients, combined with *Enterococcus faecalis* in one patient. One patient had *Citrobacter braagi* along with *Enterococcus faecalis*.
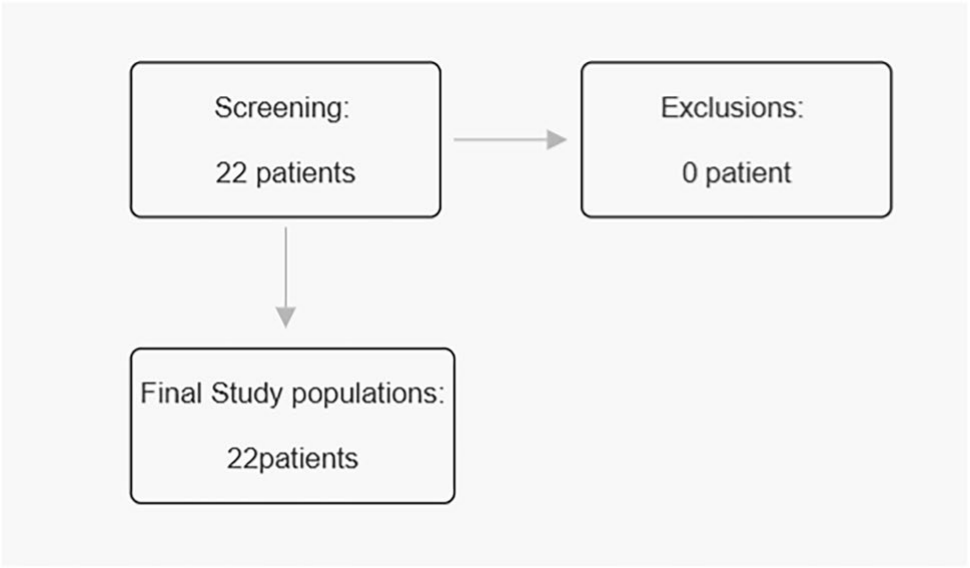


Before DRC and CEVUS, all 22 patients had normal bladder function, assessed by uroflowmetry in seven patients and 4-h voiding observation in 15. Median bladder volume was 85.4% (IQR 64.8–110.1%) of expected bladder capacity (EBC) and median residual urine was 7.3% (IQR 4.1–11.4%) of EBC. All children enroled completed both imaging studies.

Baseline renal and urinary tract imaging findings were reviewed prior to study inclusion. Apart from duplex collecting systems, no consistent pattern of hydronephrosis or hydroureteronephrosis was observed across the cohort.

### Primary outcome

**Fig. 1:**
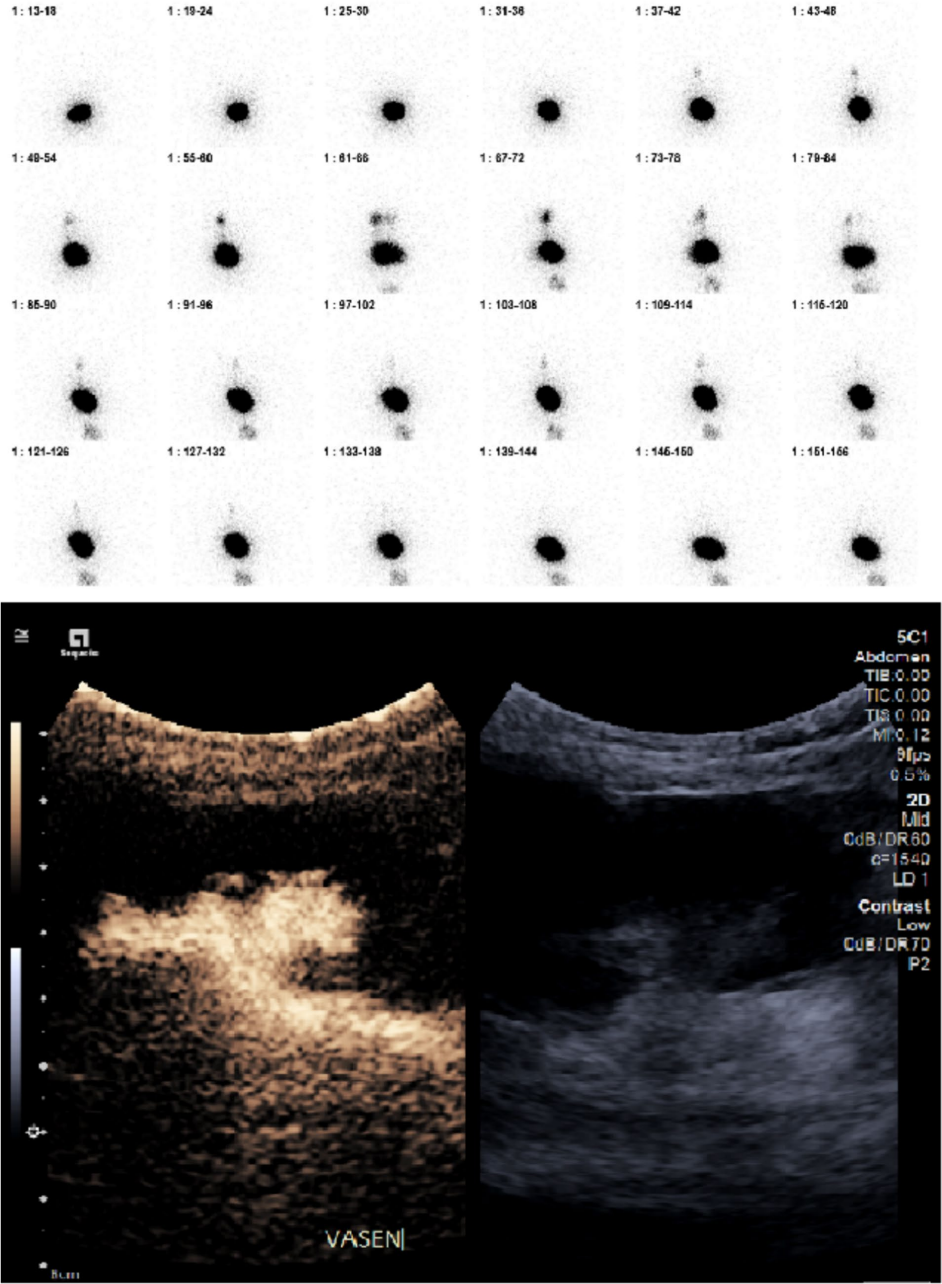
Examination of a 1-year-old child showing CEVUS and DRC study where grade 4 VUR on the left side occurred during the filling and voiding phase

At patient level, DRC and CEVUS identified VUR in 72.7% and 77.3% of patients respectively (Fig. 1), with comparable paired detection rates (exact McNemar *p* = 1.00). At renal unit level VUR was detected in 50.0% and 52.3% of renal units by DRC and CEVUS respectively, with moderate inter-modality agreement (Cohen’s *k* = 0.59; 95% CI 0.35–0.83).

DRC detected right-sided VUR in five (31.3%), left-sided in seven (43.8%), and bilateral in five patients (31.3%). CEVUS detected right-sided VUR in six (37.5%), left-sided in five (31.3%), and bilateral in five patients (31.3%). Based on CEVUS grading, the median VUR grade was 2.0 (range 2–4) on the left and 2.5 (range 2–4) on the right.

Bladder filling at first VUR detection (expressed as %EBC). Using Koff-derived EBC, the median bladder filling volume at first VUR detection was 48.0% (IQR 30.0–81.0) for DRC (*n* = 17) renal units with available first-detection volumes) and 80.5% (IQR 52.8–95.5) for CEVUS (*n* = 16). Among renal units with paired first-detection volumes available for both modalities (*n* = 13), the corresponding medians were 37.27% (IQR 26.67–82-42) for DRC and 66.67% (IQR 52.94–87.27) for CEVUS, with no statistically significant paired difference (Wilcoxon signed-rank test *p* = 0.158).

Under the study protocol using physiological bladder filling volumes and using DRC as the reference comparator, CEVUS demonstrated a sensitivity of 81.8% (95% CI 61.5–92.7) and a specificity of 77.3% (95% CI 56.6–89.9). Positive predictive value (PPV) was 78.3% (95% CI 58.1–90.3), and negative predictive value (NPV) was 81.0% (95% CI 60.0–92.3). The respective positive and negative likelihood ratios were 3.60 and 0.24.

**Fig. 2:**
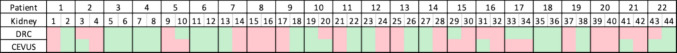
Comparison of diagnostic findings between DRC and CEVUS for the detection of VUR. The heatmap displays results for 44 renal units across 22 patients. Green indicates a positive finding (presence of reflux), and red indicates a negative finding (absence of reflux)

DRC detected VUR in 4 renal units (9.1%) that were negative on CEVUS, while CEVUS detected VUR in 5 renal units (11.4%) that were negative on DRC (Fig. 2). All discrepant findings were non-dilating reflux (grade 2). IRR was not detected on CEVUS at physiological bladder filling volumes and not assessed by DRC.

### Secondary outcome

None of the patients had hypersensitivity to the investigational products. One patient developed a UTI following the examinations. Following identification of VUR, treatment adhered to EAU guidelines. Three patients underwent endoscopic Deflux®-treatment following recurrent UTIs despite continuous prophylactic antimicrobial therapy. One patient with VUR grades 2 and 4, experienced failure of prophylactic antimicrobial therapy due to allergy. Another patient with a history of renal abscess was treated endoscopically despite bilateral grade 2 VUR. The third patient with VUR grades 3 and 4 had failed antimicrobial therapy due to non-compliance. One year later, she was diagnosed with bladder-bowel dysfunction (BBD). All other patients had uneventful outcomes within the constraints of the study follow-up.

Data on bladder filling volume at the time of VUR detection during the CEVUS study are missing for one patient. Otherwise, complete data were available for all patients.

## Discussion

In this prospective, comparative study with blinded investigators, we demonstrated that CEVUS proved comparable to direct DRC in detecting VUR, using physiological bladder filling volumes. The methods showed moderate agreement (Cohen’s *k* = 0.59) with no statistically significant difference in detection rates (McNemar *p* = 1.00). These findings support CEVUS as a radiation-free alternative for pediatric VUR diagnostics, while providing diagnostic accuracy avoiding excessive bladder filling.

VCUG is the standard diagnostic tool for VUR but involves ionizing radiation and catheterization. DRC was introduced to reduce radiation, yet recent VCUG protocol optimizations have lowered its effective dose, sometimes below that of DRC. However, effective radiation dose is highly dependent on local protocols, equipment, tracer activity, and examination duration. At our institution, the median effective dose for DRC (0.073 mSv) exceeded that of contemporary VCUG [10]. This does not imply that DRC inherently delivers higher radiation than VCUG but rather highlights that dose comparisons are institution- and protocol-specific and should be regularly reassessed. While the observed dose corresponds to approximately 5 days of background radiation in Finland and is therefore marginal, it still adds to cumulative exposure. CEVUS avoids radiation entirely and has demonstrated sensitivity and specificity comparable to both VCUG and DRC [12–18, 20, 21, 26, 27]. Despite these advantages, clinical use has remained limited, partly due to a technical learning curve [28]. The American Food and Drug Administration approved intravesical use of UCAs for pediatric imaging in 2016 and the European Medicines Agency in 2017 [29]. After approval, the use of CEVUS has steadily increased in pediatric ureteral reflux imaging. CEVUS is encouraged by the Urogenital Task Force of the European Pediatric Radiology Society as the preferred study method whenever feasible [30] VCUG remains an option, especially in preoperative assessment and for evaluating urethral pathology.

VUR is historically classified into five grades according to Heikel and Parkkulainen [25]. However, in clinical practice, this grading is often simplified into dilating vs. non-dilating VUR [31]. Dilating VUR (grades III–V) is considered clinically relevant due to higher risk of pyelonephritis, renal scarring, hypertension and long-term renal damage [32, 33]. Non-dilating VUR, on the other hand, often resolves spontaneously without aggressive intervention [31]. Therefore, it is crucial to avoid diagnostic practices likely to induce clinically irrelevant non-dilating VUR. IRR, defined as the retrograde flow of urine flows from the renal calyces into the tubulointerstitial space, has been particularly linked to renal scarring [20] and reflux nephropathy when occurring in conjunction with UTIs [34]. Detecting IRR is clinically significant and may influence treatment decisions to prevent long-term renal damage. IRR was actively assessed during CEVUS examinations but was not detected in any patient in this cohort. IRR is generally considered uncommon in low-grade reflux, being primarily associated with high-grade VUR and collecting system dilatation. Consequently, the present study permits no conclusions regarding the comparative ability of CEVUS and DRC to detect IRR. The absence of IRR in our cohort likely reflects the study population characteristics, including recurrent UTIs with confirmed normal bladder function and a predominance of non-dilating reflux. Although earlier studies [21, 34–38] have reported IRR detection with CEVUS in selected populations with higher-grade VUR, this was not observed in the current cohort.

In all diagnostic imaging for VUR, contrast material is introduced into the bladder via a urinary catheter. As bladder capacity in children is age dependent, estimating EBC is essential to avoid excessive filling. While some studies suggest that children with VUR may have unexpectedly larger bladder size [39], our study compared measured bladder volumes to EBC to ensure appropriate filling. Guerra et al. [23] showed that diagnostic VCUG is prone to bladder overfilling, possibly inducing iatrogenic reflux and potential over-diagnosis or over-treatment. Moreover, overfilling also increases the risk of complications, including rare instances of bladder rupture. To mitigate these risks, diagnostic methods should ideally detect VUR under physiological bladder filling conditions. CEVUS, when performed with physiological bladder filling volumes, addresses these concerns by avoiding ionizing radiation exposure and minimizing the risk of overfilling, while still providing detailed anatomical information.

Both DRC and CEVUS detected VUR using physiological bladder filling volumes. To account for physiological variability, stress-related effects, and the intermittent nature of reflux, two separate filling–voiding cycles were performed for each modality, in line with standard VCUG practice. Notably, comparative studies have not reported bladder filling volumes in detail [26, 27], and actual filling volumes are rarely specified in the existing DRC and CEVUS literature. Despite the uncertain clinical significance of this observation, our findings suggest that CEVUS performs comparably to DRC in identifying VUR without necessitating excessive bladder filling. In addition to diagnostic accuracy, CEVUS avoids radiation exposure and costs one-third of DRC. Regarding safety, no hypersensitivity reactions to the contrast agents were observed, and only one patient developed a UTI following the procedures. Contrasting findings between DRC and CEVUS were observed in nine renal units, all involving non-dilating (grade 2) reflux. VUR is known to be intermittent, and despite same-day examinations and standardized filling protocols, differences in timing, voiding dynamics, or transient ureterovesical junction competence may influence reflux detection. Moreover, DRC is sensitive to minimal tracer activity, whereas CEVUS provides real-time visualization and may be more dependent on the temporal coincidence of reflux with imaging. All discordant cases involved non-dilating reflux, which is generally considered of limited clinical significance and is managed conservatively. Consequently, these discrepancies would not have altered clinical management in the present cohort; treatment decisions were guided by recurrent infections, reflux grade, and overall clinical context rather than isolated detection of low-grade reflux. This supports the clinical comparability of the two modalities despite minor diagnostic discrepancy at the lower end of the grading spectrum.

## Strengths and limitations

Strengths include the prospective design, blinded interpretation and same-day paired comparison using a single catheterization. Although the bladder was emptied between modalities and two filling-voiding cycles were performed, a small order effect cannot be excluded. Physiological filling was standardized using EBC, which may not capture individual variation in bladder capacity.

The study is limited by a smaller-than-planned sample size; recruitment was halted after an interim analysis showing that the effective dose of DRC in our institution exceeded that of optimized VCUG protocols, raising ethical concerns regarding continued radiation exposure [10]. Accordingly, the study may be underpowered to detect small differences. Although baseline radiological findings, including congenital urinary tract anomalies, were reviewed, our study was not designed to evaluate correlations between VUR and prior imaging findings such as hydronephrosis, hydroureteronephrosis, or renal scintigraphy results. The number of patients with structural anomalies was small, precluding meaningful statistical comparisons. Thus, generalizability is limited to children with normal bladder function and recurrent UTIs; children with neurogenic bladder, bladder–bowel dysfunction, or major anomalies were excluded. Larger confirmatory studies are warranted.

## Conclusion

CEVUS detects VUR with sensitivity comparable to DRC using physiologic bladder filling volumes. It avoids the radiation burden, provides detailed anatomical information and is cost-effective. These advantages support CEVUS as a valuable first-line diagnostic tool for pediatric VUR.

## Data Availability

All data supporting the findings of this study are available within the paper and its Supplementary Information.

## Abbreviations

VUR: Vesicoureteral reflux
UTI: Urinary tract infections
VCUG: Voiding cystourethrography
DRC: Direct radionuclide cystography
CEVUS: Contrast-enhanced voiding ultrasonography
IRR: Intrarenal reflux
